# Glucocorticoids promote apoptosis of proinflammatory monocytes by inhibiting ERK activity

**DOI:** 10.1038/s41419-018-0332-4

**Published:** 2018-02-15

**Authors:** Adrian Achuthan, Ahmad S. M. Aslam, Quyen Nguyen, Pui-Yeng Lam, Andrew J. Fleetwood, Ashlee T. Frye, Cynthia Louis, Ming-Chin Lee, Julia E. Smith, Andrew D. Cook, Moshe Olshansky, Stephen J. Turner, John A. Hamilton

**Affiliations:** 1 Department of Medicine, Royal Melbourne Hospital, The University of Melbourne, Parkville, VIC 3050 Australia; 2 Inflammation Division, The Walter and Eliza Hall Institute of Medical Research, 1G Royal Parade, Parkville, VIC 3052 Australia; 3 Research and Development Immunoinflammation, GlaxoSmithKline Medicines Research Centre, Stevenage, SG1 2NY UK; 4 Department of Microbiology, Monash University, Clayton, VIC 3800 Australia

## Abstract

Glucocorticoids (GCs) are potent anti-inflammatory drugs whose mode of action is complex and still debatable. One likely cellular target of GCs are monocytes/macrophages. The role of GCs in monocyte survival is also debated. Although both granulocyte macrophage-colony stimulating factor (GM-CSF) and macrophage-CSF (M-CSF) are important regulators of macrophage lineage functions including their survival, the former is often associated with proinflammatory functions while the latter is important in lineage homeostasis. We report here that the GC, dexamethasone, induces apoptosis in GM-CSF-treated human monocytes while having no impact on M-CSF-induced monocyte survival. To understand how GCs, GM-CSF, and M-CSF are regulating monocyte survival and other functions during inflammation, we firstly examined the transcriptomic changes elicited by these three agents in human monocytes, either acting alone or in combination. Transcriptomic and Ingenuity pathway analyses found that dexamethasone differentially modulated dendritic cell maturation and TREM1 signaling pathways in GM-CSF-treated and M-CSF-treated monocytes, two pathways known to be regulated by ERK1/2 activity. These analyses led us to provide evidence that the GC inhibits ERK1/2 activity selectively in GM-CSF-treated monocytes to induce apoptosis. It is proposed that this inhibition of ERK1/2 activity leads to inactivation of p90 ribosomal-S6 kinase and Bad dephosphorylation leading in turn to enhanced caspase-3 activity and subsequent apoptosis. Furthermore, pharmacological inhibition of GC receptor activity restored the ERK1/2 signaling and prevented the GC-induced apoptosis in GM-CSF-treated monocytes. Increased tissue macrophage numbers, possibly from enhanced survival due to mediators such as GM-CSF, can correlate with inflammatory disease severity; also reduction in these numbers can correlate with the therapeutic benefit of a number of agents, including GCs. We propose that the ERK1/2 signaling pathway promotes survival of GM-CSF-treated proinflammatory monocytes, which can be selectively targeted by GCs as a novel mechanism to reduce local monocyte/macrophage numbers and hence inflammation.

## Introduction

Glucocorticoids (GCs) are stress hormones that modulate a wide range of physiological processes, including metabolism and inflammation^1^. Exogenous GCs are very potent anti-inflammatory and immunosuppressive agents broadly used in therapy, albeit with adverse side effects associated with long-term usage^2–4^. The negative consequences of GC therapy provide an impetus for research into gaining insights into the molecular mechanisms of GC action on immune cells.

Monocytes and macrophages are key components of the immune system. It has become apparent that these populations can be quite heterogeneous and plastic depending on the milieu to which they are exposed during their activation and/or differentiation. Two cytokines that can regulate the development and function of monocyte/macrophage populations are macrophage-colony stimulating factor (M-CSF; also known as CSF-1) and granulocyte macrophage-CSF (GM-CSF)^5^. M-CSF, which circulates at high levels, is expressed in many tissues with evidence to suggest it can govern steady state macrophage numbers and be involved in resolution of an inflammation^6–9^. In contrast, GM-CSF circulates normally at low levels but has often been implicated in inflammatory/immune responses during which its levels are elevated^7–9^. Currently, GM-CSF and its receptor are being targeted widely in clinical trials to treat autoimmunity/inflammatory diseases^10^.

The potent anti-inflammatory and immunosuppressive effects of GCs are mediated mainly by its binding to a cytosolic glucocorticoid receptor (GCR). Translocated GCR can bind directly to DNA to a canonical GC response element^11^ or act indirectly by binding to other transcription factors (e.g., NF-κB and AP-1)^12^. There is a broad consensus that GCs exert their anti-inflammatory actions on monocytes/macrophages by primarily inhibiting the transcription of proinflammatory genes (e.g., TNF) possibly by suppressing the NF-κB activity^3,13^. Accumulating evidence suggest that, in addition to their ability to suppress the production of proinflammatory mediators by monocytes/macrophages, GCs can induce specific changes in cell survival, proliferation, and phagocytosis to resolve inflammation^14–18^. We have proposed that GM-CSF and M-CSF may contribute to the progression of an inflammatory reaction in part by enhancing tissue macrophage numbers by promoting their survival and/or local proliferation, for example, in the rheumatoid synovium^19^; in contrast, one of the dramatic effects of GC during rheumatoid arthritis therapy is a striking reduction of synovial macrophage numbers^20^. However, the effects of GCs on the survival of human monocytes are controversial^14,21^, warranting a detailed investigation of their regulation of survival/death signaling pathways.

In the present study, we identified a novel mechanism of GC-induced apoptosis of proinflammatory monocytes. We provide evidence that GC treatment selectively promotes the apoptosis of GM-CSF-treated monocytes by inhibiting ERK/12 phosphorylation, which in turn leads to dephosphorylation of p90RSK resulting in the pro-apoptotic activities of Bad and caspase-3. We propose that inhibition of ERK activity, leading to apoptosis of proinflammatory monocytes, is a novel mechanism by which GCs are capable of selectively downregulating inflammatory processes.

## Materials and methods

### Reagents and Abs

Reagents included human GM-CSF (R&D Systems, Minneapoilis, MN), human M-CSF (Chiron, Emeryville, CA), dexamethasone (Dex), and mifepristone (Sigma-Aldrich, St. Louis, MO). Antibodies for Western blotting were against phospho-p38 (D3F9), p38 (D13E1), phospho-JNK1/2 (81E11), JNK1/2 (9252), phospho-ERK1/2 (D13.14.4E), ERK1/2 (137F5), caspase-3 (9662), caspase-8 (D35G2), Bcl2 (50E3), Bcl-xL (54H6), Bid (2002), Bak (D4E4), Bax (D2E11), Bim (C34C5), phospho-Bad S112 (40A9), Bad (D24A9), phospho-RSK S380 (D3H11) and RSK (32D7) (Cell Signaling Technologies, Danvers, MA), and β-actin (A5316) (Sigma-Aldrich).

### Isolation and culture of human monocytes

Buffy coats were sourced ethically and their research use was in accord with the terms of the informed consents obtained by Australian Red Cross Blood Service. Human monocytes were isolated and cultured as described before^22^. Monocytes were treated simultaneously with M-CSF (5000 U/ml), GM-CSF (10 ng/ml) or Dex (100 nM), either alone or in combination for indicated time periods.

### Quantitative PCR

Total RNA was extracted using ISOLATE II RNA Mini Kit (Bioline, London, UK) and reverse transcribed using SuperScript III reverse transcriptase (Invitrogen). Quantitative PCR (qPCR) was performed using the ABI PRISM 7900HT sequence detection system (Applied Biosystems, Carlsbad, CA) and predeveloped TaqMan probe/primer combinations for human HLA-DOA, HLA-DPA, HLA-DPB, HLA-DQA, HLA-DQB, HLA-DRA, HLA-DRB, CIITA, TREM1, and HPRT (Applied Biosystems). Threshold cycle numbers were transformed to cycle threshold values, and the results were plotted using GraphPad Prism version 5.04.

### Microarray analyses

RNA was isolated from three independent cell preparations, each derived from a single donor as before^22,23^. Microarray analyses were preformed following recommended protocols supplied by Agilent Technologies (Santa Clara, CA, USA) as before^22^. Microarray intensity data were read using the read.maimages function from the Limma Bioconductor R package with background correction using the normexp method (https://www.bioconductor.org/packages/release/bioc/html/limma.html). Fold changes and *p* values were obtained using the RUVinv function from ruv R package^24,25^ (http://www-personal.umich.edu/~johanngb/ruv/index.html). The list of control genes was from Eisenberg et al.^26^ The data set and technical information compliant with minimum information about a microarray experiment (MIAME)^27^ can be found at the ArrayExpress Archive Web site (http://www.ebi.ac.uk/arrayexpress/; E-MTAB-2212).

### Cell viability and apoptosis assays

Human monocytes (1 × 10^6^) were cultured in 6-well BD Falcon™ adherent tissue-culture plates following various treatments at 37 °C in a humidified, 5% CO_2_ atmosphere. Cells were collected and viable cells were determined by Trypan blue exclusion method. Apoptotic cells, displaying externalized phosphatidylsterine, were measured by flow cytometry staining with a FITC-conjugated annexin V detection kit (BD Biosciences, Frankin Lakes, NJ). The percentage of apoptotic cells is determined as a ratio of annexin V positive cells, but propidium iodide (PI) negative cells, to the total number.

### Caspase-3 activity assay

Proteolytic activity of caspase-3 in lysates of monocytes was determined using the ApoTarget™ Caspase-3 Colorimetric Protease Assay kit (Invitrogen). Human monocytes (5 × 10^6^) were lysed and cytosolic extract (200 μg) was incubated at 37 °C for 2 h with 200 μM substrate, DEVD-*p*NA, is composed of the chromophore, *p*-nitroanilide (*p*NA), and a synthetic tetrapeptide, DEVD (Asp-Glue-Val-Asp), which is the upstream amino acid sequence of the caspase-3 cleavage site in poly (ADP-ribose) polymerase. Upon cleavage of the substrate by caspase-3-free *p*NA light absorbance was quantified using a microplate reader at 405 nm.

### Flow cytometry

Human monocytes were treated with treated M-CSF (5000 U/ml), GM-CSF (10 ng/ml) or Dex (100 nM), either alone or in combination for indicated periods of time. Cells were stained with fluorochrome-conjugated mAbs specific for human CD14 PerCP-Cy5.5 (M5E2), CD80 APC-H7 (L307.4), HLA-DR PE (G46-6), CD95 PE-Cy7 (DX2), CD178 PE (NOK-1), and their isotype controls (BD Biosciences, San Rose, CA) and then analyzed using a CyAn ADP analyzer (Beckman Coulter, Brea, CA).

### Western blotting

Whole-cell extracts were lysed and Western blotted as described previously^22^. Protein concentrations of the samples were determined with a Bio-Rad protein assay kit. Equal amounts of protein were loaded on 10% NuPAGE gels (Invitrogen). The separated proteins were transferred onto a polyvinylidene fluoride membrane and then Western blotted with appropriate Abs. Western blots were quantified by densitometry using Quantity One version 4.6.9 (Bio-Rad) and resulting data were plotted as bar graphs as mean ± SEM.

### Statistics

Statistical analyses between groups were performed using either Student’s paired *t*-test or one-way ANOVA Tukey post-test as indicated (GraphPad Prism 5.04). The *p* values < 0.05 indicate significance. Data were plotted as mean ± SEM from at least three independent experiments using GraphPad Prism version 5.04.

## Results

### Glucocorticoid induces apoptosis in GM-CSF-treated monocytes

**Fig. 1:**
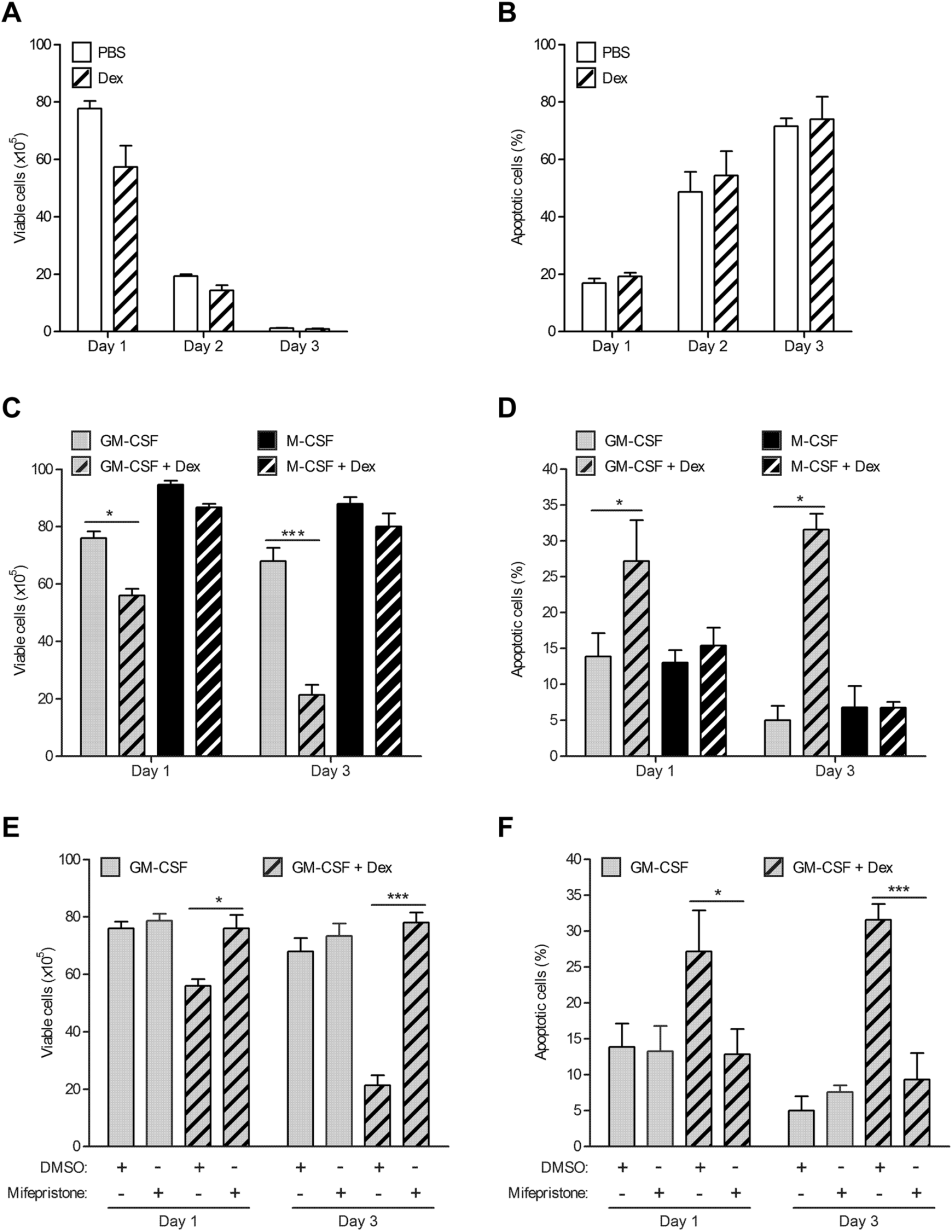
Glucocorticoid induces apoptosis in GM-CSF-treated monocytes but not in M-CSF-treated monocytes. **a**,** b** Human monocytes (1 × 10^6^) were cultured in the absence (PBS) or presence of dexamethasone (Dex) (100 nM) over three days. **a** Viable cells **b** apoptotic cells by trypan blue exclusion method and Annexin V staining, respectively. **c**, **d** Human monocytes (1 × 10^6^) were cultured with either GM-CSF (10 ng/ml) alone, M-CSF (5,000 U/ml) alone or together with Dex over three day period. **c** Viable cells **d** apoptotic cells were determined. **a**–**d** Experiments with the same donors. **e**, **f** Human monocytes (1 × 10^6^) were pre-treated with mifepristone (1 μM) for 30 min before culture in either GM-CSF alone or together with Dex over three day period. **e** Viable cells **f** apoptotic cells were determined. Graphs were plotted as mean ± SEM (*N* = 4). Statistical analyses were performed using Student’s paired *t*-test, where *p < 0.05 and ***p < 0.001

Human monocytes were treated with 100 nM dexamethasone (Dex) or PBS over 3 days. Viable and apoptotic cells were assessed by the Trypan blue exclusion method and annexin V staining, respectively. A representative dot plot illustrating the gating strategy is provided in Supplementary Figure S1. The number of viable cells dropped significantly in both PBS and Dex-treated groups, but no significant difference in viable cell numbers was observed between the groups (Fig. 1a). Correlating with the decrease in viable cells, the apoptotic cell numbers increased over the three day period (Fig. 1b). Again, no significant difference in apoptotic cell numbers was observed between the groups over this period.

To assess whether the presence of a pro-survival factor would still maintain the survival of monocytes in the presence of Dex, monocytes were treated with GM-CSF (10 ng/ml) or M-CSF (5,000 U/ml), either alone or together with Dex for 1 and 3 days. After day 1, GM-CSF and Dex co-treated monocytes showed some increased cell death compared to monocytes treated with GM-CSF alone, an effect not observed with M-CSF and Dex co-treatment (Fig. 1c). These differences were even more pronounced after day 3. Consistent with the decreasing numbers of viable cells, GM-CSF and Dex co-treated cells underwent apoptosis in increased numbers compared to monocytes treated with GM-CSF alone (Fig. 1d). On the other hand, no difference in annexin V staining was observed between monocytes co-treated with M-CSF and Dex and with M-CSF alone.

To demonstrate that the Dex-induced apoptotic effect in GM-CSF-treated monocytes is mediated via the glucocorticoid receptor (GCR), the steroid receptor antagonist, mifepristone was utilized. Pre-treatment of monocytes with 1 µM mifepristone for 30 min before stimulation with either GM-CSF alone or together with GM-CSF and Dex resulted in abrogation of GC-induced apoptosis in GM-CSF-treated monocytes (Fig. 1e, f), an effect that lasted for at least 3 days.

### Glucocorticoid inhibits GM-CSF-induced dendritic cell maturation and TREM1 signaling pathways

Given the striking difference noted above in the effects of Dex on the pro-survival action of the two CSFs and given the transcriptional role of the GCR, a whole-genome-wide transcriptomic analysis was carried out for monocytes undergoing the various treatments for 16 h. Microarrays of human monocytes treated with Dex found 685 differentially expressed genes, with 183 upregulated genes and 502 downregulated genes (Table S1). GM-CSF treatment of monocytes resulted in 150 (90 upregulated, 60 downregulated) differentially expressed genes (Table S2), whereas M-CSF treatment resulted in 373 (30 upregulated, 343 downregulated) differentially expressed genes (Table S3). Strikingly, among the genes regulated by Dex, GM-CSF, and M-CSF treatments, only eight genes were regulated by all three conditions suggesting distinct gene expression profiles (Table S4). Detailed functional analyses of these differentially expressed genes, using ingenuity pathway analysis (IPA) tool, are also provided (Tables S1–3).

**Fig. 2:**
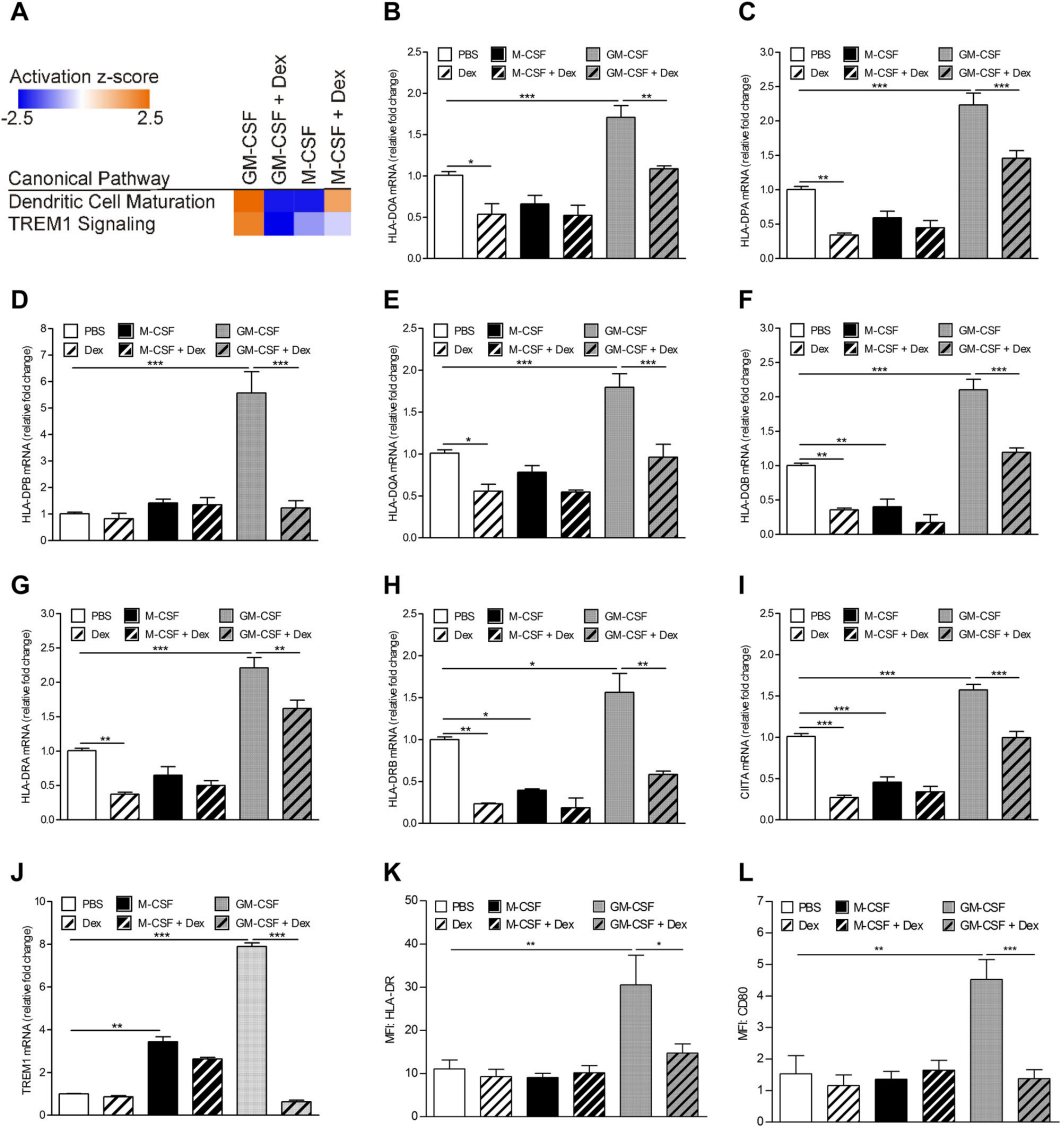
Glucocorticoid inhibits GM-CSF-regulated dendritic cell maturation and TREM1 signaling pathways. Human monocytes (1 × 10^6^) were treated with either dexamethasone (Dex) (100 nM), M-CSF (5000 U/ml), GM-CSF (10 ng/ml), or as a combination of Dex with each CSF for 16 h. Isolated RNA was subjected to **a** gene expression arrays, showing significantly regulated canonical pathways among the treatment groups by ingenuity pathway comparison analysis. **b**–**j** qPCR with HPRT RNA as reference. mRNA expression, assayed in triplicate, was plotted relative to that at 16 h in culture medium containing vehicle (PBS), which was given an arbitrary value of 1.0. FACS analysis of cell surface expression of **k** HLA-DR and **l** CD80. The graphs represent mean ± SEM (*N* = 4). Statistical analyses were performed using one-way ANOVA with Tukey’s multiple comparison test, where *p < 0.05, **p < 0.01, and ***p < 0.001

To understand further the effects of Dex on GM-CSF-treated and M-CSF-treated monocytes, microarray analyses of human monocytes co-treated with either GM-CSF and Dex or M-CSF and Dex were performed. GM-CSF and Dex co-treatment resulted in 190 (142 upregulated, 48 downregulated) differentially expressed genes (Table S5). On the other hand, microarray analysis of human monocytes co-treated with M-CSF and Dex found 328 (270 upregulated, 58 downregulated) differentially expressed genes (Table S6). Furthermore, only 55 genes were commonly regulated among the GM-CSF + Dex and M-CSF + Dex co-treatment groups (Table S4). Strikingly, all of the commonly regulated genes were regulated in the same direction in the co-treatments. Significantly, IPA comparison analysis of the transcriptomes revealed that dendritic cell maturation and triggering receptor expressed on myeloid cells 1 (TREM1) signaling pathways were activated by GM-CSF, whereas GM-CSF + Dex co-treatment robustly inhibited both pathways; these data are in contrast to M-CSF and M-CSF + Dex co-treated monocytes (Fig. 2a).

Interestingly, among the number of commonly regulated genes between Dex and M-CSF a number of MHCII molecules were found to be downregulated (Table S4). Furthermore, upstream analysis of these two transcriptomes (Tables S1 and S3), identified the MHCII master regulator, CIITA, as also being downregulated. The genes identified by microarray analysis, as well as related MHCII molecules, were validated by qPCR (Fig. 2b–i). In agreement with the microarray data, a general pattern of Dex and M-CSF suppressing MHCII molecules was observed. Of potential relevance to the dendritic cell maturation finding by the IPA comparison analysis, GM-CSF upregulated all of the MHCII genes measured, as well as CIITA (Fig. 2b–i). Strikingly, all of these genes were significantly downregulated by Dex when monocytes were co-treated. Also, GM-CSF upregulated HLA-DR and CD80 cell surface expression, which were downregulated by Dex (Fig. 2k, l).

TREM1 receptor signaling has an important role in the amplification of inflammation and cell survival, which are dependent on ERK activity^28^. Although GM-CSF and M-CSF were both found to upregulate TREM1 expression, Dex significantly downregulated it only when monocytes were co-treated with GM-CSF and Dex (Fig. 2j).

### Glucocorticoid inhibits GM-CSF-activated ERK1/2 phosphorylation which correlates with increased caspase-3 activity

**Fig. 3:**
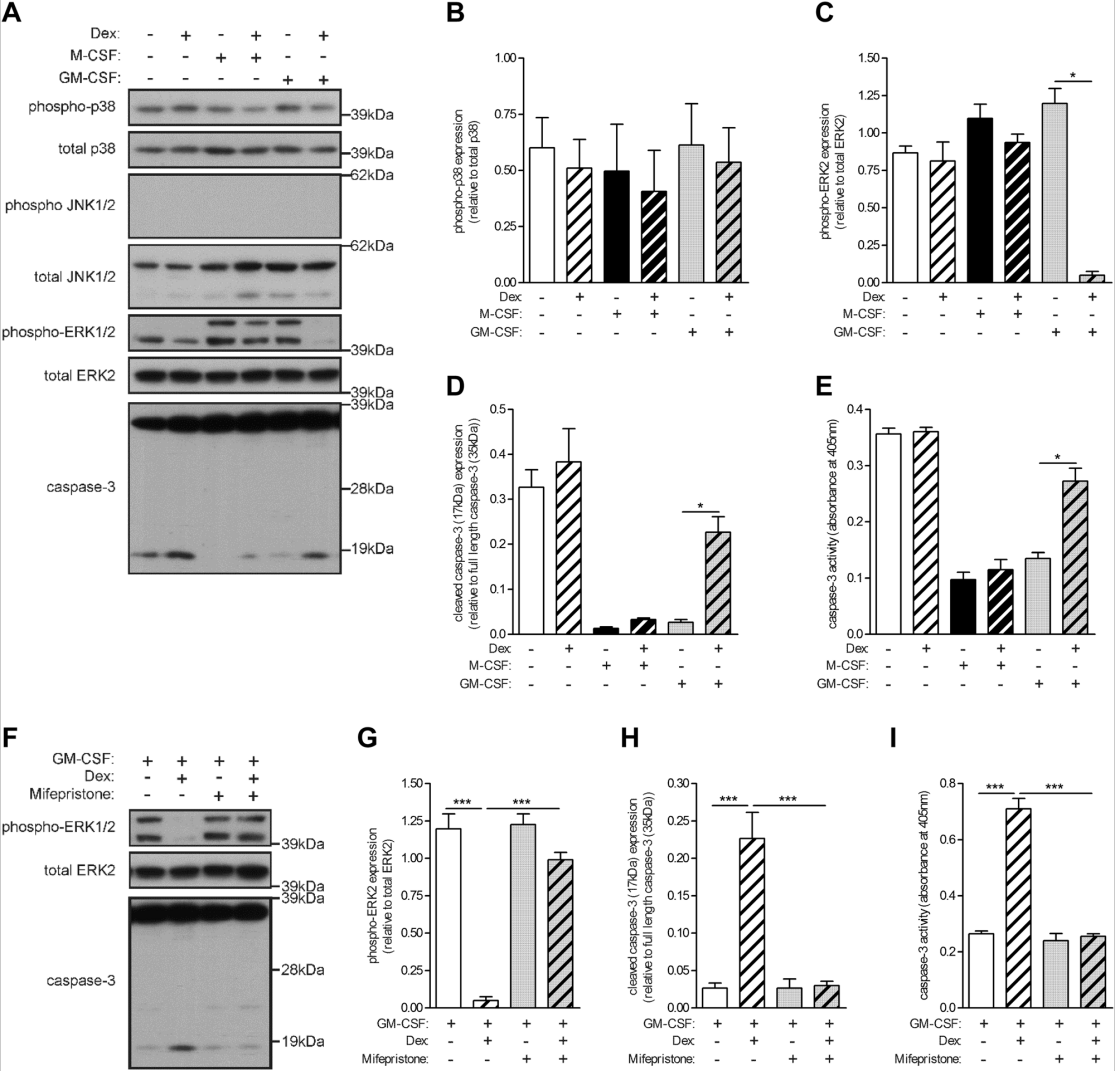
Glucocorticoid inhibits GM-CSF-activated ERK1/2 phosphorylation, which is accompanied by increased caspase-3 activity. **a**–**e** Human monocytes (1 × 10^6^) were treated with either dexamethasone (Dex) (100 nM), M-CSF (5000 U/ml), GM-CSF (10 ng/ml), or as a combination of Dex with each CSF for 16 h. **a** Whole-cell lysates were subjected to Western blotting with anti-phospho-p38, anti-p38, anti-phospho-JNK, anti-JNK, anti-phospho-ERK1/2, anti-ERK2, and anti-caspase-3 Abs. **b**–**d** Quantified data of activated/cleaved protein relative to total/full-length protein, as indicated **e** Caspase-3 activity. **f**–**i** Human monocytes (1 × 10^6^) were pre-treated with mifepristone (1 μM) for 30 min before treated with either GM-CSF alone or together with Dex for 16 h. **f** Whole-cells lysates were subjected to Western blotting with anti-phospho-ERK1/2, anti-ERK2 and anti-caspase-3 Abs. **g**, **h** Quantified data of activated/cleaved protein relative to total/full-length protein, as indicated **i** Caspase-3 activity. **a**–**i** Experiments from the same donors. Graphs were plotted as mean ± SEM (*N* = 3). Statistical analyses were performed using one-way ANOVA with Tukey’s multiple comparison test; *p < 0.05 and ***p < 0.001

The activation states of different members of the mitogen-activated protein kinase (MAPK) family are known to modulate monocyte survival and death^29^. Also, ERK activity is required for dendritic cell maturation^30^ and TREM1-mediated macrophage survival^28^, the two pathways arising out of the IPA comparison analysis above for the GM-CSF + Dex vs GM-CSF treatments. Based on these analyses, to determine whether GCs can regulate the activation of MAPKs to promote apoptosis in GM-CSF-treated monocytes, human monocytes were treated with Dex, M-CSF and GM-CSF alone, or as a combination of Dex with each CSF, for 16 h. Whole-cell lysates were then subjected to Western blotting with antibodies specific for phosphorylated p38 MAPK, JNK1/2 and ERK1/2. Basal phosphorylation of p38 MAPK was detected in human monocytes but there was no change in its expression across the different treatments (Fig. 3a, b); also there was no activation of JNK1/2 by Dex, M-CSF nor GM-CSF (Fig. 3a). No changes were detected in the levels of JNK1/2 and p38 MAPK. ERK2 (p42) was basally activated in human monocytes, but its activity could be increased somewhat following M-CSF and GM-CSF treatment (Fig. 3a). On the other hand, ERK1 (p44) was activated only in the presence of either M-CSF or GM-CSF. M-CSF-induced ERK1 activation was not affected by GC; interestingly, there was a significant decrease in GM-CSF-induced ERK1, as well as basal ERK2 phosphorylation, when monocytes were co-treated with GM-CSF and Dex (Fig. 3a, c). Total ERK1/2 expression did not change following the different treatments. Significantly, ERK1/2 activity inversely correlated with the proteolytically cleaved active form of caspase-3 (Fig. 3a, d). The increased levels of the active form of caspase-3 detected by Western blotting was further validated by a colorimetric caspase-3 activity assay (Fig. 3e). Notably, we neither detected any caspase-8 activity nor measured any differential cell surface expression of the death receptor molecules, CD95 and CD178, under the various treatments of monocytes (data not shown).

In order to confirm that GC-regulated ERK1/2 deactivation in GM-CSF-treated monocytes was mediated via the GCR, mifepristone was again utilized. Pre-treatment of monocytes with mifepristone restored ERK1/2 phosphorylation in GM-CSF and Dex co-treated monocytes (Fig. 3f, g). Significantly, this restored ERK1/2 activation correlated with a striking decrease in the active cleaved form of caspase-3 (Fig. 3f, h). Furthermore, the caspase-3 activity in monocytes pre-treated with mifepristone before stimulating with GM-CSF and Dex co-treatment was also decreased to the lower basal level found in GM-CSF-treated monocytes (Fig. 3i).

### Glucocorticoid inhibits GM-CSF-induced Bad phosphorylation

**Fig. 4:**
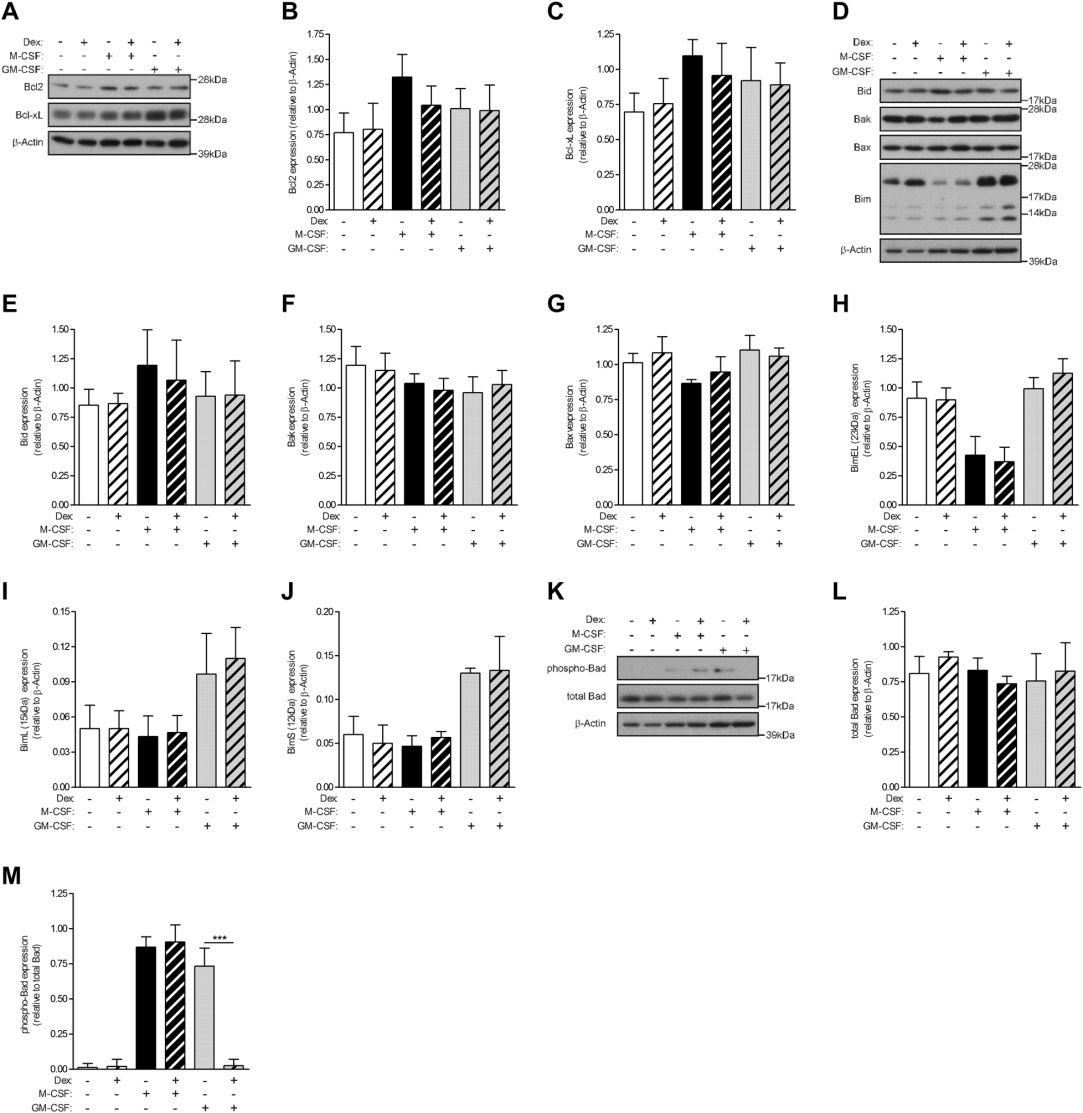
Glucocorticoid inhibits GM-CSF-induced Bad phosphorylation. Human monocytes (1 × 10^6^) were treated with either dexamethasone (Dex) (100 nM), M-CSF (5000 U/ml), GM-CSF (10 ng/ml), or as a combination of Dex with each CSF for 16 h. Whole-cell lysates were subjected to Western blotting with antibodies against **a** anti-apoptotic proteins **d** pro-apoptotic proteins **k** Bad phosphorylation. **b**, **c**, **e**–**j**, **l**, **m** Quantified data of activated/apoptotic protein relative to total/β-actin protein, as indicated. Graphs were plotted as mean ± SEM (*N* = 3). Statistical analyses were performed using one-way ANOVA with Tukey’s multiple comparison test; ***p < 0.001

The Bcl2 family consists of a number of evolutionarily conserved proteins containing the Bcl2 homology domain that regulates apoptosis through control of mitochondrial membrane permeability and release of cytochrome *C*^31^. Levels of both anti-apoptotic and pro-apoptotic proteins were measured in order to determine if Dex can regulate their expression to promote apoptosis selectively in GM-CSF-treated monocytes. Expression levels of the anti-apoptotic members, Bcl2 and Bcl-xL, did not change under the different treatments (Fig. 4a–c). Similarly, the levels of the pro-apoptotic family members, Bid, Bak, and Bax, did not change under the various treatments (Fig. 4d–g); the levels of the three isforms of another family member, Bim, namely BimEL (23 kDa), BimL (15 kDa), and BimS (12 kDa), did not change with GM-CSF treatment, either alone or with Dex, but, intriguingly, BimEL levels were lower in M-CSF-treated monocytes, regardless of the absence or presence of Dex (Fig. 4d, h–j). The lack of differentially expressed apoptotic proteins under the various treatments of monocytes was in agreement with the absence of differentially expressed apoptotic genes from the microarray analyses above. Bad is another member of the Bcl2 family, but its pro-apoptotic activity is regulated through its phosphorylation^32^. Bad phosphorylation was not detected basally but measurable in monocytes treated with M-CSF and GM-CSF alone, as well as with M-CSF + Dex (Fig. 4k–m). In contrast, Bad activity was not detected in GM-CSF + Dex co-treated monocytes. Again, total Bad expression did not vary under the different treatment conditions.

### Glucocorticoid-mediated inhibition of ERK1/2 activity leads to Bad dephosphorylation and apoptosis of GM-CSF-treated monocytes

**Fig. 5:**
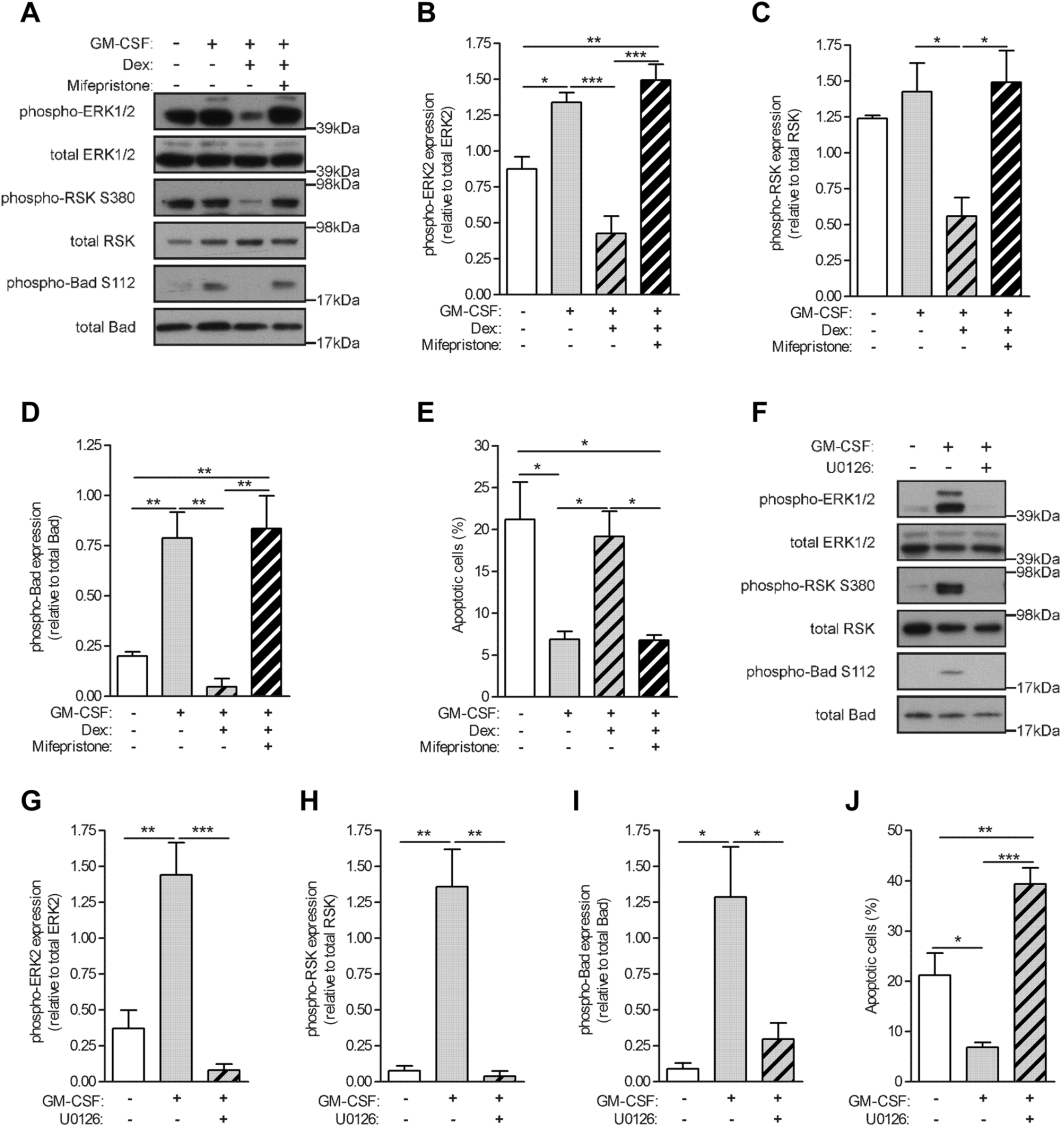
Glucocorticoid-mediated inhibition of ERK1/2 activity leads to Bad dephosphorylation and monocyte apoptosis. **a**–**e** Human monocytes (1 × 10^6^) were pre-treated with mifepristone (1 μM) or DMSO for 30 min before treatment with GM-CSF (10 ng/ml) alone or together with dexamethasone (Dex) (100 nM) for 16 h. **a** Whole-cell lysates were subjected to Western blotting with anti-phospho-ERK1/2, total ERK1/2, phospho-RSK, total RSK, phospho-Bad, or total Bad Abs. **b**–**d** Quantified data of activated protein relative to total protein, as indicated. **e** Apoptotic cells by Annexin V staining. **f**–**j** Human monocytes (1 × 10^6^) were pre-treated with MEK inhibitor, UO126 (10 μM), or DMSO for 30 min before treatment with GM-CSF (10 ng/ml) for 16 h. **f** Whole-cell lysates were subjected to Western blotting with anti-phospho-ERK1/2, total ERK1/2, phospho-RSK, total RSK, phospho-Bad, or total Bad Abs. **g**–**i** Quantified data of activated protein relative to total protein, as indicated. **j** Apoptotic cells by Annexin V staining. Graphs were plotted as mean ± SEM (*N* = 3). Statistical analyses were performed using one-way ANOVA with Tukey post-test; *p < 0.05, **p < 0.01, and ***p < 0.001

Growth factors, such as M-CSF and GM-CSF, can activate specific anti-apoptotic kinases, which can then modulate the activity of apoptotic proteins such as Bad^33^. Furthermore, the 90 kDa ribosomal-S6 kinase (RSK), a downstream effector in the MAPK signaling cascade, has been shown to induce Bad phosphorylation^34^. As Dex selectively inhibited ERK1/2 activation in GM-CSF-treated monocytes, we investigated whether such inhibition could lead to Bad dephosphorylation and the subsequent increase in apoptosis observed above. Human monocytes were treated with GM-CSF alone or together with Dex for 16 h, in the absence or presence of a prior treatment (30 min) with mifepristone. As noted earlier, monocytes had a basal ERK2 activation (Fig. 5a). Treatment with GM-CSF led to enhanced activation of both ERK1/2 (Fig. 5a, b). Co-treatment of GM-CSF and Dex resulted in a significant decrease in this ERK1/2 phosphorylation that can be restored by pre-treating monocytes with mifepristone. Correlating with the decreased ERK1/2 activity, there were lower levels of RSK phosphorylation in GM-CSF + Dex co-treated monocytes (Fig. 5a, c). This decrease in RSK activity was also restored in monocytes pre-treated with mifepristone. Correlating with the modulation of the ERK1/2 and RSK activities, Bad activity was detected in monocytes treated with GM-CSF alone or pre-treated with mifepristone before being stimulated with both GM-CSF and Dex together (Fig. 5a, d). Significantly, the increased Bad activation strongly correlated with a decrease in apoptotic monocytes (Fig. 5e).

In order to support a role for ERK1/2 in GM-CSF-induced Bad phosphorylation and subsequent survival of human monocytes, the specific MEK inhibitor, U0126, was used. As expected, pre-treatment of monocytes with 10 μM U0126 before treating with GM-CSF resulted in a robust inhibition of the GM-CSF-stimulated ERK1/2 activity (Fig. 5f, g). This inhibition of ERK1/2 activity also led to an abrogation of both RSK and Bad activities (Fig. 5f, h, i). Importantly, inhibition of ERK1/2 activity resulted in the enhanced apoptosis of GM-CSF-treated monocytes (Fig. 5j).

## Discussion

GCs were first used to treat patients with rheumatoid arthritis in 1949 and have become the most common therapy for many inflammatory disorders. They remain very effective immunosuppressive and anti-inflammatory agents used in the treatment of many autoimmune and allergic diseases, as well as to prevent allograft rejection^35^. However, long-term use of GCs is associated with adverse side effects that restrict their clinical application. Even though their various modes of action are still debated, it is broadly accepted that GCs mediate their anti-inflammatory effects in part by downregulating the proinflammatory functions of monocytes/macrophages^36^. By co-treating human monocytes with either GM-CSF or M-CSF in conjunction with Dex, we propose a molecular mechanism as to how GCs can selectively induce apoptosis of GM-CSF-treated monocytes while not affecting the survival of M-CSF-treated counterparts.

Human monocytes have a relatively short life-span, with a half-life in the blood stream of around 1–2 days before migrating into tissues or dying spontaneously by apoptosis^37^. Similarly, in the absence of a stimulus monocytes undergo apoptosis when cultured in vitro. In agreement with previous reports^38–40^, we did not find that Dex itself could promote the survival of monocytes in vitro as almost all of the Dex-treated monocytes died after 3 days in culture similar to those in culture medium alone. Apoptosis of monocytes is prevented by addition of the hematopoietic growth factors, M-CSF and GM-CSF. Strikingly, Dex triggered differing survival effects when co-treated with the CSFs: GM-CSF and Dex co-treated monocytes underwent gradual cell death over 3 days, whereas M-CSF and Dex co-treated monocytes survived over this period. Significantly, the inhibition of GCR activity by mifepristone prevented the Dex-induced cell death in the monocytes co-treated with GM-CSF and Dex, suggesting that the Dex-induced apoptosis occurs through transcriptional mechanisms.

We investigated above the molecular mechanisms underlying this GC-induced differential survival effects on GM-CSF-treated and M-CSF-treated monocytes. A whole-genome-wide transcriptome analysis was utilized to begin to uncover these mechanisms. The microarray analysis revealed quite distinct gene expression profiles for Dex-, GM-CSF-, and M-CSF-treated monocytes. The microarray data were analyzed by IPA to gain insights into the biologically meaningful molecular interactions and to identify functionally related gene networks. For only the GM-CSF-treated monocytes, microarray analysis identified dendritic cell maturation and TREM1 signaling pathways as being suppressed by Dex, both of which are, at least in part, controlled by MAPKs^28,30^. This prior analysis led us to monitor MAPK activities and we demonstrated that, among the MAPKs, activation of ERK1 was not detected in PBS, Dex, and GM-CSF and Dex co-treated monocytes; however, it was observed in GM-CSF-treated and M-CSF-treated monocytes. In the presence of Dex, M-CSF, but not GM-CSF, was able to maintain ERK1/2 activity and promoted cell survival, which were reflected in the lower levels of caspase-3 activity. Significantly, inhibiting GCR activity in the GM-CSF and Dex co-treated cultures resulted in restoration of ERK1/2 activity and the subsequent decrease in caspase-3 activity. Caspase-3 is downstream of caspase-8 and caspase-9, which are the initiating caspases of the extrinsic and intrinsic caspase cascade, respectively^41^. We were unable to detect caspase-8 activity and also failed to measure any increase in cell surface expression of the death receptor molecules, CD95 and CD178, in GM-CSF and Dex co-treated monocytes, suggesting that Dex-induced apoptosis of GM-CSF-treated monocytes is by an intrinsic apoptotic pathway. Consistent with these findings, Dex has been shown not to have any effects on dendritic cell CD95 and CD178 expression^42^.

**Fig. 6:**
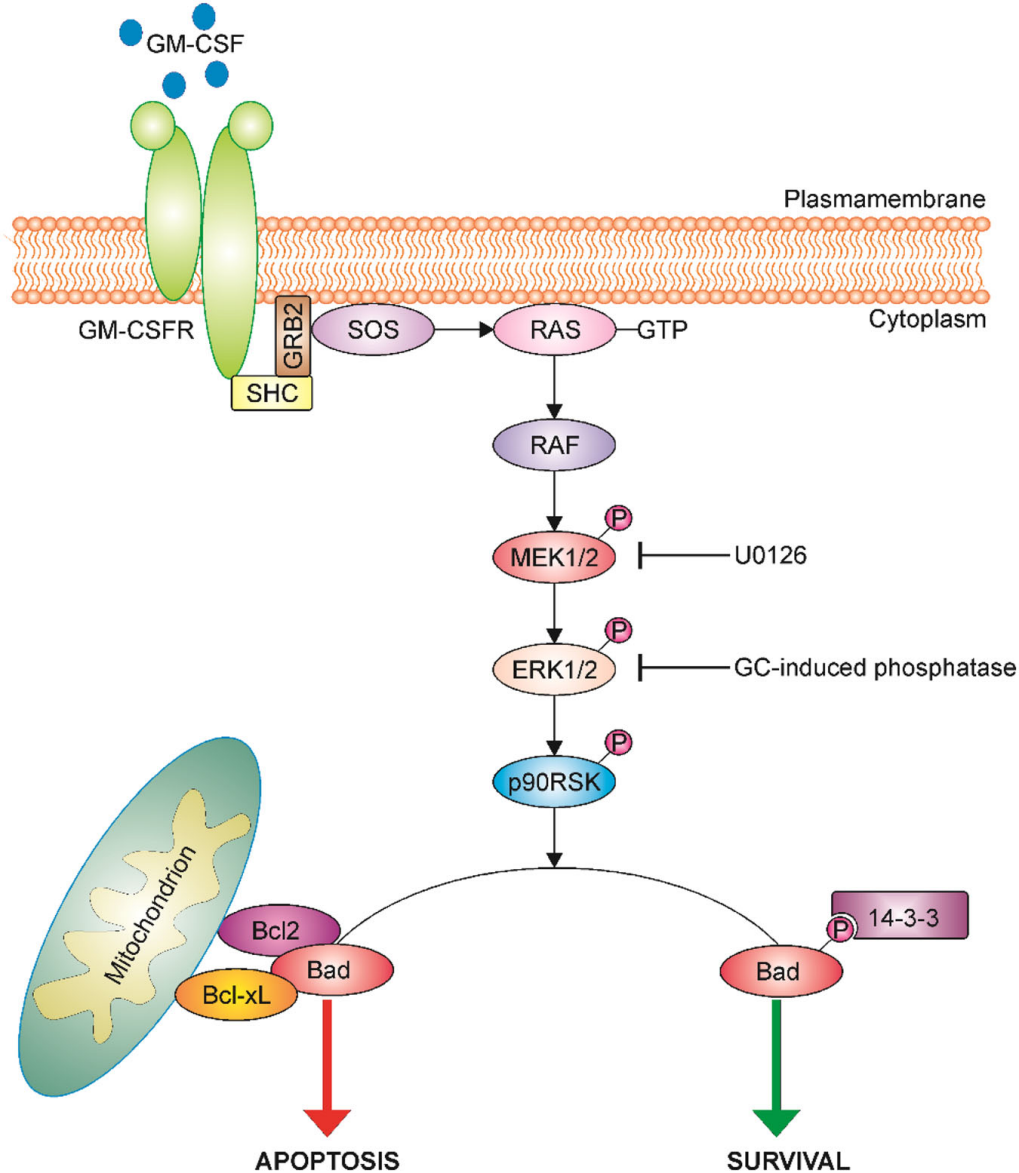
Proposed glucocorticoid-induced apoptotic signaling pathway in GM-CSF-treated monocytes. A schematic diagram illustrating a possible GM-CSF-induced ERK1/2 signaling pathway in monocytes which is inhibited by a GC leading to Bad deactivation and subsequent apoptosis. GM-CSF binding to its receptor activates a Raf/MEK/ERK signaling cascade leading in turn to ribosomal associated p90RSK kinase activation. The pro-apoptotic Bad protein is phosphorylated by p90RSK and subsequently binds to the cytoskeletal scaffold protein, 14-3-3. In the absence of a growth factor or upon inhibition of the Raf/MEK/ERK pathway by the pharmacological inhibitor (U0126) or a GC-induced phosphatase(s), p90RSK remains inactivated and is unable to phosphorylate Bad. Non-phosphorylated Bad in turn can interfere with the anti-apoptotic proteins, Bcl-xL and Bcl2, resulting in loss of mitochondrial membrane integrity and ultimately apoptosis

Bcl2 family proteins are key regulators of apoptosis that can function as cell death antagonists (Bcl2, Bcl-xL, and Mcl-1) or agonists (Bid, Bak, Bax, and Bim)^43^. Protein expression of the family members examined did not show any differential expression when monocytes were treated with growth factor alone or together with Dex, suggesting that GCs might be regulating their activity rather than expression in monocytes. BimEL expression was lower in M-CSF-treated monocytes, regardless of the absence or presence of Dex, compared to its expression in PBS-treated or GM-CSF-treated monocytes. Bim isoforms have been suggested to have distinct roles in apoptosis—for example, BimEL and BimS promote intrinsic cell death, whereas BimL is required for the formation of degradative autolysosomes^44^. Bad is also a member of the Bcl2 family; however, its pro- or anti-apoptotic function is usually controlled by its phosphorylation status. The 90 kDa ribosomal-S6 kinase (p90RSK), a downstream effector in the MAPK signaling cascade, inactivates Bad by phosphorylation at serine 112. Phosphorylated Bad is sequestered in the cytosol by binding to 14-3-3^45,46^. When not phosphorylated, Bad induces apoptosis by interacting with the anti-apoptotic Bcl2-family members, Bcl-xL and Bcl2, thereby allowing two other pro-apoptotic proteins, Bak and Bax, to aggregate on the mitochondrial membrane and induce release of cytochrome *C*, followed by caspase activation and apoptosis^31^. It is generally believed that survival factors induce activation of specific anti-apoptotic kinases, which modulate the activity of Bad^47^. Indeed, we show here that both M-CSF and GM-CSF by themselves can activate the Raf/MEK/ERK signaling cascade that results in Bad phosphorylation and monocyte survival. Co-treatment of monocytes with M-CSF and Dex also sustained ERK1/2 and Bad activities, as well as monocyte survival. However, co-treatment of monocytes with GM-CSF and Dex resulted in ERK1/2 deactivation leading to Bad dephosphorylation, the subsequent increase in caspase-3 activity and eventually apoptosis (Fig. 6). The putative phosphatase(s) responsible for dephosphorylating ERK1/2 specifically in GM-CSF and Dex co-treated monocytes remains to be determined. Several other studies have identified a number of GC-induced phosphatases that are capable of dephosphorylating MAPK family members. For example, GC-mediated attenuation of ERK1/2 phosphorylation in fibroblasts is regulated through DUSP1 expression^48^.

In addition to promoting survival, the role of GM-CSF in stimulating human blood monocytes for increased antigen-presenting functions is widely reported^49,50^. IPA analysis and subsequent validation of MHCII molecule expression confirmed also the ability of GCs to suppress antigen-presenting pathways as previously documented^16, 51,52^, possibly forming part of their immunosuppressive and anti-inflammatory role. GCs may thus be acting on GM-CSF-treated monocytes and suppressing their maturation into a potent antigen-presenting cell. The expression of MHCII molecules is controlled by the master transcription regulator, CIITA. Of potential mechanistic relevance, GM-CSF treatment of human monocytes increased the amount of CIITA associated with HLA-DR promoter^49^. In addition, our microarray and subsequent qPCR analyses revealed that both M-CSF and Dex downregulated CIITA expression, which may contribute to the decreased MHCII gene expression in the monocytes treated with either of these two factors with implications for dendritic cell maturation.

In summary, what is notable from our data is that GM-CSF-treated monocytes and not the M-CSF-treated cells gradually die in the presence of a GC. We have delineated above a molecular mechanism which is proposed to account for this GC-mediated apoptosis. We provide evidence that, by selectively inhibiting the Raf/MEK/ERK signaling pathway, GCs induce apoptosis of GM-CSF-treated monocytes, while failing to do so in M-CSF-treated monocytes. Macrophage numbers in inflamed tissues can correlate with disease severity, for example, in the rheumatoid synovium, and one mechanism could be enhanced survival^19^; increased macrophage survival is considered to be one possibility for the proinflammatory action of GM-CSF^7,8,10,19^. We speculate that the selective GC-mediated apoptosis in GM-CSF-treated monocytes could be a mechanism for preventing excessive inflammation by eliminating monocytes/macrophages that are responding locally to a proinflammatory stimulus. Interestingly, in this context, in rheumatoid arthritis patients the benefit of a number of therapeutic agents, including GCs, correlates with a reduction in synovial tissue macrophage numbers^20^. The selective GC-induced apoptosis of proinflammatory monocytes/macrophages may thus have clinical significance

## Electronic supplementary material

Supplemental Figure S1

Supplemental Table S1

Supplemental Table S2

Supplemental Table S3

Supplemental Table S4

Supplemental Table S5

Supplemental Table S6
