# Supplementary material for: Glucocorticoids promote apoptosis of proinflammatory monocytes by inhibiting ERK activity
Source: Cell Death Dis. 2018 Feb 15;9(3):267. doi: 10.1038/s41419-018-0332-4 (PMC5833693; doi:10.1038/s41419-018-0332-4)
Supplement: Supplementary file 1 — Supplemental Figure S1 [file 41419_2018_332_MOESM1_ESM.docx]

**Supplementary Figure S1**

| **A**   | |
| --- | --- |
|  | |
| **B**   | **C**   |
|  | |

**Figure S1: Glucocorticoid induces apoptosis in GM-CSF-treated monocytes but not in M-CSF-treated monocytes**

**(A)** Human monocytes (1x10^6^) were cultured in the absence (PBS) or presence of dexamethasone (Dex) (100 nM) over a three day period. A representative dot plot indicating the percentage of live (annexin V negative and propidium iodide (PI) negative), apoptotic (annexin V positive and PI negative) and dead (annexin V positive and PI positive) cells at day 1. **(B)** Human monocytes (1x10^6^) were cultured with either GM-CSF (10 ng/ml) alone, M-CSF (5,000 U/ml) alone or together with Dex over a three day period. A representative dot plot indicating the percentage of live, apoptotic and dead cells at day 1. **(C)** Human monocytes (1x10^6^) were pre-treated with mifepristone (1 μM) for 30 minutes before culture in either GM-CSF alone or together with Dex over a three day period. A representative dot plot indicating the percentage of live, apoptotic and dead cells at day 1. The dot plots are representative of four independent experiments, which are quantified in Figure 1.
